# Differences in blastomere totipotency in 2-cell mouse embryos are a maternal trait mediated by asymmetric mRNA distribution

**DOI:** 10.1093/molehr/gaz051

**Published:** 2019-09-04

**Authors:** E Casser, S Wdowik, S Israel, A Witten, S Schlatt, V Nordhoff, M Boiani

**Affiliations:** 1 Max Planck Institute for Molecular Biomedicine, Muenster, Germany; 2 Core Genomic Facility, University Hospital Muenster, Muenster, Germany; 3 Centre for Reproductive Medicine and Andrology, University Hospital Muenster, Muenster, Germany

**Keywords:** blastomere, *Cops3*, epiblast, FISH (fluorescent *in situ* hybridization), totipotency, transcriptome

## Abstract

It is widely held that the first two blastomeres of mammalian embryos are equally totipotent and that this totipotency belongs to the group of regulative properties. However, this interpretation neglects an important aspect: evidence only came from successful monozygotic twins which can speak only for those pairs of half-embryos that are able to regulate in the first place. Are the frequently occurring incomplete pairs simply an artefact, or do they represent a real difference, be it in the imperfect blastomere’s ability to regulate growth or in the distribution of any compound X that constrains regulation? Using the model system of mouse embryos bisected at the 2-cell stage after fertilization, we present evidence that the interblastomere differences evade regulation by external factors and are already latent in oocytes. Specifically, an interblastomere imbalance of epiblast production persists under the most diverse culture conditions and applies to the same extent in parthenogenetic counterparts. As a result, cases in which twin blastocysts continued to develop in only one member account for 65 and 57% of zygotic and parthenogenetic pairs, respectively. The interblastomere imbalance is related to the subcellular distribution of gene products, as documented for the epiblast-related gene *Cops3*, using mRNA FISH in super-resolution mode confocal microscopy. Blastomere patterns of *Cops3* mRNA distribution are α-amanitin-resistant. Thus, the imbalance originates not from *de novo* transcription, but from influences which are effective before fertilisation. These data expose previously unrecognized limits of regulative capacities of 2-cell stage blastomeres and point to aspects of cytoplasmic organization of the mouse oocyte that segregate unequally to blastomeres during cleavage.

## Introduction

The overwhelming impression gained from the literature is that mammalian totipotency belongs to the group of the regulative properties, and that the first two blastomeres of the classically studied mammalian embryo, the mouse embryo, are equally totipotent: each is ascribed with the ability to generate a new organism via the primary germ layers (trophectoderm, primitive endoderm and epiblast (EPI)). A conceptual loom to weave totipotency and regulation and to give these words a meaning is the one provided by Edwards and Beard: ‘Cellular totipotency might be better understood as a cellular response to external stimuli during specific developmental stages’ and helps ‘to clarify several aspects of regulation’ (Edwards and Beard, 1997). One such response is that mounted after the bisection of 2-cell stage embryos. The products are called monozygotic (MZ) twins regarding their origin and half-embryos with respect to their biomass, and they are generally regarded as a proof of equal blastomere totipotency. A variant of the bisection experiment has been performed previously in which 2-cell embryos were reconstructed by exchanging one of the two blastomeres with one from another 2-cell embryo (cross-reconstitution; Motosugi *et al.*, 2005). Had the blastomeres not been equally totipotent, then the cross-reconstitution in a proportion of cases would have matched two blastomeres that were defective in the same way, resulting in total impairment. None of the cross-reconstituted embryos was missing parts, for example, inner cell mass (ICM) or trophectoderm, and pups were occasionally born after blastocyst transfer to the uterus (Motosugi *et al.*, 2005). Collectively, these data supported the portrait of 2-cell blastomeres as being equal in developmental potency. The impressive ability of successful half-embryos to catch up with body weight (Papaioannou and Ebert, 1995) led to the generalization that everything else was also properly regulated.

However, this interpretation neglects an important aspect: anatomical completeness and regulation of body weight can speak only for those half-embryos that are able to regulate development in the first place. When the published data are re-evaluated critically, they reveal that, upon separation, only one of the two blastomeres develops into a mouse in most cases (Tsunoda and McLaren, 1983; Togashi *et al.*, 1987; Wang *et al.*, 1997; Sotomaru *et al.*, 1998; Casser *et al.*, 2017, 2018). The singletons had been dismissed historically as exceptions to the rule, experimental errors or idiosyncrasies. At odds with these explanations, we reported that there is a reproducible EPI imbalance in ≈70% of twin blastocyst pairs derived from more than 1000 bisected 2-cell stage embryos (Casser *et al.*, 2017). Importantly, all these blastocysts had an ICM, but the ICM of one member of the pair contained no or too few EPI cells, whose absolute number seems critical for the implanted blastocyst to develop to term (Morris *et al.*, 2012). Exceptions or errors cannot possibly be so frequent (≈70%), making it hard to relinquish alternative explanations for the 2-cell blastomere imbalance. Among these explanations, it has been proposed that sister blastomeres may possess incomplete regulative abilities because the early embryo is not totally free of order and, specifically, the blastomeres are not free of developmental order, even from the beginning (Zernicka-Goetz, 2005). Therefore, we wanted to elucidate whether the observed interblastomere difference in totipotency is due to imperfect regulation of potentiality, as opposed to inheritance of a differing degree of potentiality from the oocyte.

In the present study, 2-cell stage blastomeres were separated and exposed to various culture media as external stimuli that elicit more or less regulation, as per the definition above (Edwards and Beard, 1997). Under the provisions of regulative development, a blastomere exposed to different culture conditions should disclose a competence to switch from epigenetically determined cell fate paths and escape constraints that would otherwise lead to an EPI imbalance. We found a behaviour of 2-cell blastomeres which would not have been expected if they had been equally totipotent and equally able to regulate development, namely: conservation of unequal EPI contribution, no matter whether regulation thrived under superior embryo culture conditions or languished under the limitations imposed by parthenogenesis. Unequal EPI contributions could be rooted in any kind of molecules (e.g. proteins, RNAs and lipids). Exemplarily, we show that this feature appears related to the spatial distribution of an mRNA, *COP9 signalosome complex subunit 3* (*Cops3;*Yan *et al.*, 2003), in the 2-cell embryo, as revealed by mRNA FISH in super-resolution mode confocal microscopy (Raj *et al.*, 2008). This mRNA was shown to be essential for EPI maintenance and proliferation in peri-implantation mouse embryos (Yan *et al.*, 2003). The FISH intensity distribution of *Cops3* mRNA is more eccentric, relative to the geometric centre, than that of *Gapdh*; it is also more eccentric than the mRNA distribution of *Pou5f1* (*Oct4*), a gene previously linked to totipotency (Pesce and Schöler, 2000) albeit relinquished later on (Wu *et al.*, 2013). Thus, we hereby provide molecular correlates of EPI formation imbalance between sister blastomeres. Our data do not question that the regulative capacity of the mouse embryo is generally very pronounced, but it shows that this capacity is limited regarding EPI-mediated totipotency. Hence, our study demonstrates the limits of regulative capacities of the 2-cell stage blastomeres and reconsiders the segregation of developmentally relevant factors that become unequally distributed to the first two blastomeres, indicating that this could possibly be due to hitherto unknown aspects of the architecture of oocyte cytoplasmic organization.

## Materials and Methods

### Compliance with regulations on research animals

All mice used were maintained in individually ventilated type 2 L cages in the animal facility of the MPI Münster, with a controlled temperature of 22°C, a 14/10 h light/dark photoperiod and free access to water and food (Harlan Teklad 2020SX). Procedures used in this study followed the ethical guidelines of the European Laboratory Animal Science Associations (FELASA) and the ARRIVE reporting guidelines (Kilkenny *et al.*, 2010). On the local regulatory level, mice were used for experiments according to the ethical approval issued by the Landesamt für Natur, Umwelt und Verbraucherschutz (LANUV) of the state of North Rhine-Westphalia, Germany (Permit number 84-02.04.2016.A229).

### Collection of oocytes and embryo production

Six- to eight-week-old B6C3F1 females were primed with 10 I.U. each of pregnant mare serum gonadotropin (PMSG, Intergonan, Intervet) and human chorionic gonadotropin (hCG, Ovogest, Intervet) injected intraperitoneally 48 h apart at 5 p.m., and then killed to collect unfertilized metaphase II (MII) oocytes or mated to CD1 stud males (6–12 months old) to collect pronucleus-stage oocytes after cervical dislocation. Cumulus cells were removed in hyaluronidase (50 I.U./mL in Hepes-buffered Chatot, Ziomek and Bavister (CZB) medium). MII oocytes were activated in Ca-free α-minimum essential medium (Cavaleri *et al.*, 2006) containing 10 mM SrCl_2_ and 5 μM Latrunculin B (Terashita *et al.*, 2012), from which they were removed after 6 h as pronucleus-stage oocytes. Pronuclear oocytes from either parthenogenesis or fertilization were transferred to 500 μL of embryo culture medium in a 4-well plate without oil overlay, at 37°C under 6% CO_2_ in air, until further manipulation on the next day. Two-cell embryos were bisected as described (Casser *et al.*, 2017). The zona-free blastomeres were allocated individually to embryo culture in a 96-well plate with a round bottom (75 μL medium per well, without oil overlay). Culture media were potassium simplex optimization medium enriched with aminoacids (KSOM(aa)) (Ho *et al.*, 1995), made in house, or commercially available SAGE 1-step, CSC, G-TL, GLOBAL and GM501 (Morbeck *et al.*, 2017). Blastomeres were cultured for an additional 72 h at 37°C under 6% CO_2_ in air until the blastocyst stage. Pronucleus-stage oocytes were cultured in the presence of RNA polymerase II-III inhibitor, α-amanitin (100 μg/mL; Levey and Brinster, 1978) to assess the role of embryonic transcription in the spatial distribution of mRNA in the 2-cell stage.

### Analysis of cell lineage allocation of blastocysts

Blastocysts from intact or bisected 2-cell embryos (72 h after bisection) were analyzed by performing an immunostaining followed by confocal microscopy imaging to identify and map the different cell lineages, as described (Schwarzer *et al.*, 2012). The following primary antibodies were applied simultaneously to the specimens overnight at 4°C: anti-Cdx2 mouse IgG1κ (Emergo Europe, The Hague, The Netherlands, cat. no. CDX2–88), anti-Nanog rabbit IgG (Cosmo Bio, Tokyo, Japan, cat. no. REC-RCAB0002P-F) and anti-Sox17 goat IgG (R&D Systems, cat no. AF1924) in dilutions of 1:200, 1:2000 and 1:100, respectively. Appropriate Alexa Fluor-tagged secondary antibodies (Invitrogen) were matched to the primaries and incubated for 2 h at room temperature. Embryos were placed in 5 μL drops of PBS on a 50-mm thin-bottomed plastic dish (Greiner Bio-One, Lumox hydrophilic dish; Frickenhausen, Germany) and overlaid with mineral oil (M8410 Sigma). Images were captured on the stage of an inverted microscope (Eclipse 2000-U; Nikon, Düsseldorf, Germany) fitted with a spinning disk confocal unit (Ultra View RS3; Perkin-Elmer LAS, Jügesheim, Germany). A Nikon Plan Fluor 40X oil immersion lens (NA 1.30) was used. Twenty optical sections per blastocyst were captured using a Hamamatsu ORCA ER digital camera (Hamamatsu Photonics KK, Japan). Maximum intensity projections were analyzed with ImageJ Version 1.46j, counting the CDX2-positive, SOX17-positive and NANOG-positive cells manually.

### Embryo transfer (ET) and postimplantation development

Blastocysts from intact or bisected 2-cell embryos (72 h after bisection) were transferred as either pools of eight or single pairs (twin and co-twin, with six oocytes as carriers) to one uterine horn of pseudopregnant CD1 recipients that had the copulation plug from vasectomized CD1 males 2 days prior to the ET. The recipients generally weighed between 25 and 30 g and were older than 8 weeks but no older than 3 months. Within the same experiment, the recipients were of same age ±1 week and of same weight ± 2 g. Prior to surgery, CD1 recipients were anesthetized with Ketamine (80 mg/kg body weight)/Xylazine (16 mg/kg)/Tramadol (15 mg/kg) in PBS injected intraperitoneally. Embryos were delivered to the uterine lumen using a mouth-operated, flame-polished glass pipette through a hole made in the cranial region of the uterine horn using a 27G hypodermic needle. Wounds in the skin were closed with metal clips. The surgery *per se* took typically 10–15 min per mouse. The post-surgery recovery area was warmed to ≈30°C using infrared lamps. Animals were returned to their cages when fully awake. Pregnancies were recorded by caesarean section shortly prior to the natural term (embryonic day (E) 17.5).

### Extended in-vitro *c*ulture (IVC) of blastocyst to a stage equivalent to an egg cylinder

Zona-free twin blastocysts were seeded as pairs in gelatin-coated, flat-bottomed 96-well plates containing 150 μL IVC1 medium per well. Culture took place at 37°C under 6% CO_2_ in air. After 3 days when the blastocysts had adhered to the bottom, IVC1 was carefully aspirated and replaced with IVC2 medium (Bedzhov *et al.*, 2014).

### Transcriptome analysis of single blastomeres and single blastocysts

Total RNA was extracted using the ZR RNA Microprep Kit (Zymo Research Corporation, Irvine, USA) without the DNase digestion step. Gene expression profiling was performed using Affymetrix GeneChip® Mouse Transcriptome Array 1.0 (Affymetrix United Kingdom Ltd, High Wycombe, United Kingdom) containing < 214 000 transcripts. The fragmented and biotinylated DNA targets were prepared according to the standard Affymetrix WT Pico Reagent Kit protocol (Affymetrix GeneChip® WT Pico Reagent Kit) using 11 amplification cycles from the total RNA starting material available. GeneChips were hybridized, washed and stained in the Affymetrix Fluidics Station 450, according to the standard GeneChip Expression Wash, Stain and Scan protocol (Affymetrix GeneChip Wash, Stain and Scan Kit). Hybridization took place at 45°C for 16 h. The GeneChips were scanned using the Affymetrix 3000 7G scanner. The Affymetrix Expression Console and Transcriptome Analysis Console Version 4.0.1.36 from Applied Biosystems was used for the microarray data analysis. The ‘Signal Space Transformation Robust Multiarray’ averaging method was applied for background correction, normalization and probe summarization. Gene expression differences were determined by applying an analysis of variance.

### mRNA FISH of oocytes and 2-cell embryos in super-resolution mode confocal microscopy

We followed the protocol of Jansova and colleagues (Jansova *et al.*, 2018) with modifications as follows. Samples were washed for 5 min in nuclease-free PBS (Sigma Aldrich; D8662), fixed for 20 min in freshly prepared fixation buffer (3.8% (v/v) RNA-grade formaldehyde (Sigma Aldrich; F8775) in nuclease-free PBS) and transferred directly into permeabilization and storage buffer (70% (v/v) nuclease-free ethanol (Roth GmbH; 9065.5) in 0.1% dimethyl dicarbonate-treated (Fluka; 40 130) Milli-Q water) for at least 12 h at 4°C. Storage for up to 4 weeks without sample degradation has been demonstrated. Immediately before hybridization, samples were rehydrated and calibrated in 0.1% Triton X-100 (Carl Roth; 3051.2) supplemented wash buffer (10% (v/v) deionized formamide in 2X SSC buffer) for at least 15 min at 37°C. The subsequent hybridization was performed in Stellaris® RNA FISH probes containing hybridization buffer (10% (w/v) dextran sulfate (Sigma Aldrich; D8906), 10% (v/v) deionized formamide in 2X SSC buffer) at 37°C overnight. After hybridization, all samples were washed twice in wash buffer for 30 min each at 37°C, counterstained with DAPI (Sigma Aldrich; D9542) and re-buffered in 2X SCC solution at room temperature. All work were carried out in uncoated round-bottomed 96-well plates (Nunc; 163 320) with free-floating samples, which were transferred using a mouth-operated pipette with a bent and flame-polished tip. Hybridized samples were pooled in 4 μL droplets of Vectashield mounting medium (Vector Laboratories; H-1000) on CELLview™ culture dishes (Greiner; 627 861) overlaid with mouse embryo-grade mineral oil (Sigma Aldrich; M8410) for imaging. A microinjection of *Cops3* mRNA in one blastomere of the 2-cell stage embryo was performed for positive controls. An incubation period of 1 h at 37°C with RNase A (Thermo Fisher Scientific; AM2270) in PBS prior to washing and hybridization was performed for negative controls. The samples treated with RNase were subsequently incubated according to the steps mentioned above. Commercially available Stellaris® RNA FISH probes against mouse *Gapdh* (BioSearch Technologies; VSMF-3015-5), *Oct4* (BioSearch Technologies; VSMF-3067-5) and custom-designed probes against mouse *Cops3* (NCBI gene ID: 26572) utilizing the BioSearch Technologies design service, both labelled with Quasar® 670, were used for hybridization. Final stock solutions, re-buffered in TE buffer pH 8.0 (Thermo Scientific; AM9849), of 12.5 μM aliquoted in 3 μL units were stored at −30°C until use in a final working concentration of 125 nM.

### Analysis of the image eccentricity after mRNA FISH in super-resolution mode confocal microscopy

Images were acquired from a Zeiss laser-scanning microscope LSM 880 with Airyscan utilizing the super-resolution mode. A Zeiss Plan-Apochromat 63x (1.40 N.A.) oil DIC M27 objective in addition to a 1.8-fold magnification was used to generate z-stacks of 512 × 512-pixel resolution of the largest cross sections of each sample. The 633 nm HeNe laser line allowed the extinction of the probe fluorophore, while the 405 nm diode laser line did the same for the DAPI signal. Specific setup settings included a pixel dwell time of ∼ 4 μs and separated z-stack acquisitions for both channels separately, always with 633 nm first and a slice interval of 0.22 μm. Raw images were later processed using Airyscan deconvolution of the Zeiss ZEN Software 2.3 (Release Version 14.0.0.0). Maximum intensity projections of 10 slices (corresponding to 2.22 μm in total) taken in the equatorial region were generated using Fiji/ImageJ (Version 2.0.0-rc-69/1.52i) for image analysis. All 2-cell stage embryos were oriented alike, transforming the projections so that the long axis of the blastomeres was parallel to the y-axis of the image frame and shifted 90° in respect of the x-axis. Fitting regions of interest (drawn precisely around blastomeres or approximated as circles around whole embryos or oocytes) were then measured for all parameters with the built-in analysis tool of Fiji/ImageJ. The native images (red channel) were normalized using identical settings for contrast and brightness for reasons of comparability before being used for analysis. Images were pseudocoloured for demonstration (not for analysis!) purposes (Antczak and Van Blerkom, 1997, 1999; Schulz and Roberts, 2011). A LUT colour palette was used, which was adapted from the ‘Union Jack’ LUT of Fiji/ImageJ.

In order to harness the fine distribution of the mRNA FISH signal, we took inspiration from the analysis of Park and colleagues (Park *et al.*, 2012). We reasoned that in the case of a uniform signal, there is overlap between all x and y coordinates of the pixels that compose the image (centroid) and the intensity-weighted average of the x and y coordinates of those pixels (centre of mass); whereas, in the case of a non-uniform signal, the centre of mass of the FISH signal departs from the centroid of the same image. Therefore, the transcript pattern can be related to the distance (eccentricity) between the two average positions (centroid and centre of mass) using the Pythagorean theorem: the longer the distance is, the longer the hypotenuse of the triangle constructed, using delta x and delta y as legs of the right angled triangle.

### Graphical data rendering and statistical analysis

The values presented are mean value ± standard deviation. Diagrams were generated in the R program, except for the constellation diagrams (JMP). Blastocyst and birth rates, cell counts, interblastomere ratios and eccentricity values were analyzed using non-parametric tests in the statistical program JMP (SAS). Microarray data analysis was performed in-house using the Excel output of Transcriptome Analysis Console Version 4.0.1.36 from Applied Biosystems/Thermo Fisher Scientific.

**Figure 1:**
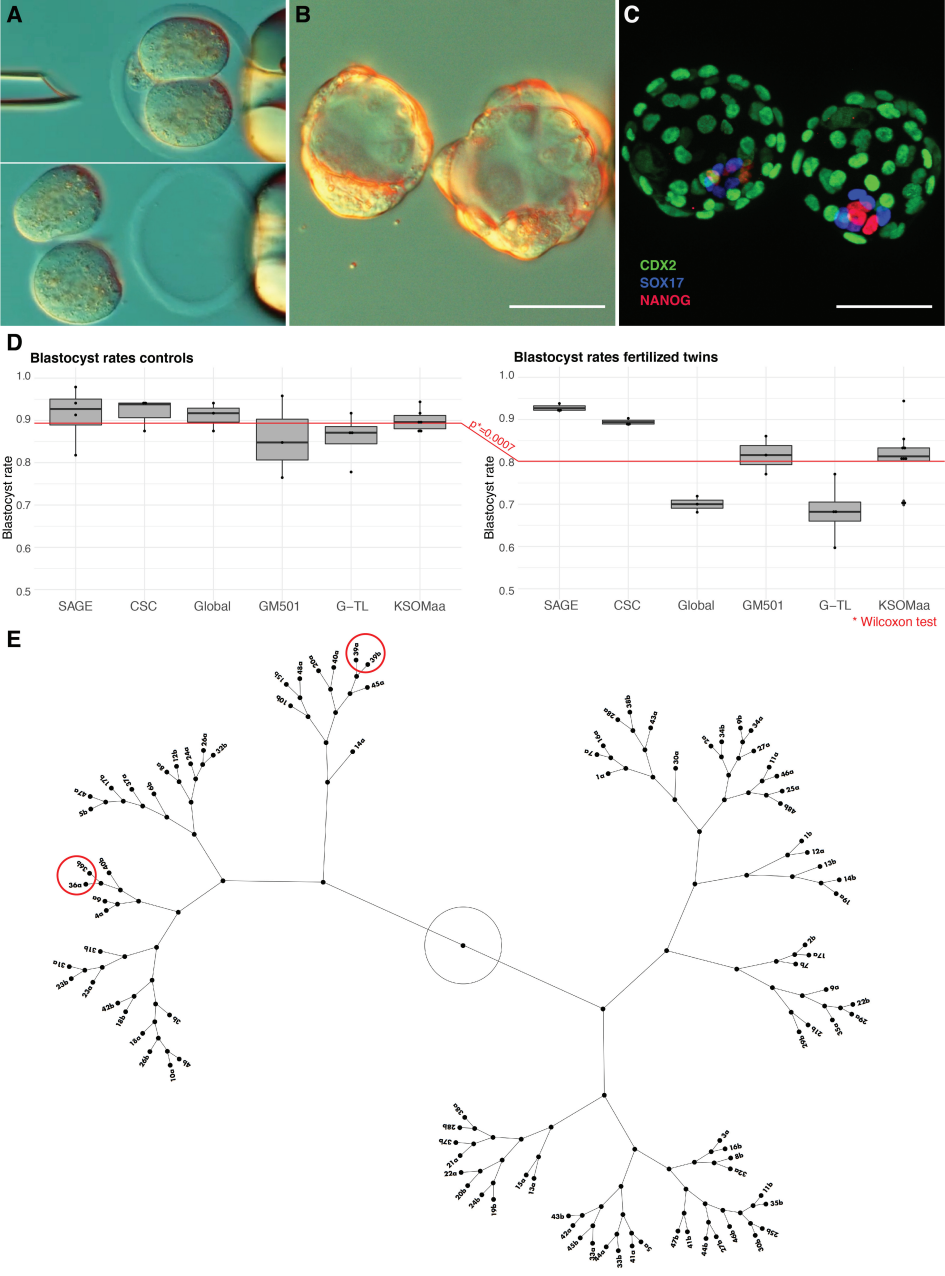
**The model system of bisected 2-cell mouse embryos and its pre-validation to test the regulative versus non-regulative origin of totipotency imbalance.** (**A**) In the course of 1624 bisections, we found that both blastomeres survived in 1584 cases (97.5 ± 2.0%) and only one blastomere survived in the remaining cases. The blastomeres damaged during bisection lysed either immediately or during the next 20–30 min and could be easily distinguished and discarded. (**B**) Under individual embryo culture conditions, which are needed to prevent the twin and co-twin from fusing back together, the separated blastomeres developed further when they were cultured zona-free in 96-well plates using potassium simplex optimization medium enriched with aminoacids (KSOM)(aa), SAGE 1-step, GM501, G-TL, CSC or Global medium. Size bar, 50 μm. (**C**) The blastocysts all had a morphological ICM, but in most monozygotic pairs the cell counts in the primary germ layers differed in the twin and co-twin. Size bar, 50 μm. (**D**) Blastocyst rates of twins were high in all groups albeit slightly below the control rates (control = intact, non-bisected embryos). Total starting numbers of embryos in (D): SAGE, 102 controls, 156 twins; CSC, 83 controls, 15 twins; Global, 81 controls, 118 twins; GM501, 111 controls, 136 twins, G-TL, 104 controls, 166 twins; KSOM(aa), 231 controls, 540 twins. (**E**) Using the cell counts of the three germ layers as well as their sum, statistical clustering analysis returned the correct pair associations in a minority of twin blastocyst pairs, as shown for KSOM(aa) as an example.

### Data accessibility

In compliance with MIAME guidelines (Brazma *et al.*, 2001), the microarray data discussed in this publication have been deposited in the NCBI’s Gene Expression Omnibus (Edgar *et al.*, 2002) and are accessible through Gene Expression Omnibus. GSE120905: adaptive molecular responses of mouse half-embryos grown in different culture media (GM501 Cult, SAGE 1 step, KSOM(aa)) (Supplementary Table SI). GSE111589: single-cell analysis of sister blastomeres of parthenogenetic 2-cell mouse embryos respecting pair associations (Supplementary Table SIII). GSE110599: analysis of gene expression during mouse preimplantation development *in vitro*, with or without a background of exogenous ovarian stimulation (data for Supplementary Fig. S4).

## Results

### Developmental performance of 2-cell stage blastomeres does not respond to physical separation but to culture conditions

In order to elucidate whether developmental differences between sister mouse blastomeres (Casser *et al.*, 2017) are due to imperfect regulation of potentiality or the inheritance of a differing degree of potentiality from the oocyte, it is essential to show upfront that (i) regulation can be harnessed experimentally and (ii) interblastomere differences are not a latent effect of cell damage inflicted during the separation. Blastomere lysis has occurred seldom (2.5%) after mechanical bisection conducted within 5 h of the first zygotic cleavage (Fig. 1A). The two zona-free blastomeres were cultured apart in the widely used KSOM(aa) medium, in which we originally showed that twin and co-twin present an EPI imbalance at the blastocyst stage (Casser *et al.*, 2017). Intact embryos served as controls. Although we confirmed that the two zona-free blastomeres would reunite if placed next to each other and would form blastocysts efficiently (homologous reconstitution, 95%, *n* = 20; cross reconstitution, 85%, *n* = 20), the careful manual repositioning of the blastomeres introduces additional micromanipulation, reducing the validity of this control. We made the twin and co-twin pairs thrive by means of culture media to determine to what extent this imbalance is amenable to regulation. We used the most refined media commercially available, which are those pretested in the ‘mouse embryo assay’ (Quinn and Horstman, 1998) for subsequent use in fertility clinics to generate human babies. We chose single-step media (SAGE 1-step, GM501, G-TL, CSC, GLOBAL) to perform continuous culture without the disturbance of medium change. Twin blastocyst formation (Fig. 1B) was high in all groups, albeit slightly below the rates of intact embryos (Fig. 1D), and the response to the media was genome wide. For example, more than 10% of the transcriptome was differently expressed (*t*-test, *P* < 0.01) in twin blastocysts cultured in GM501 and in SAGE 1step, respectively, compared to twins cultured in KSOM(aa) (deposited dataset GSE120905; Supplementary Table SI). The sum of the cells in the two halves in these twin blastocysts should not be less than the intact embryo; otherwise, it would mean that the bisection damaged one of the blastomeres. To clarify this, we performed immunostaining for the EPI marker NANOG in combination with the identification of the remaining cells (CDX2-positive trophectoderm, SOX17-positive primitive endoderm; Schwarzer *et al.*, 2012) (Fig. 1C). While twin blastocysts accumulated more cells in the superior media (45–58 cells) than in KSOM(aa) (32 cells), there was no difference in the sum of the two halves within the same medium to that of the undivided embryos (e.g. 64 ± 22 and 61 ± 16 cells in KSOM(aa), *P* = 0.406, *t*-test; in the other media *P* ≥ 0.540), attesting to the negligible effect of the bisection.

Altogether, these data show that our combined method of blastomere separation and single embryo culture does not cause latent damage that impairs cell proliferation, while the embryos respond to the new surroundings as documented by gene expression changes. Thus, our method can be applied to investigate whether these changes also make the embryos competent to leave the original path of differentiation that would otherwise lead to EPI imbalance.

### Regulation of the EPI cell number but not of the EPI cell ratio in MZ twin pairs

Embracing the totipotency definition of Edwards and Beard (1997), we delved into the germ layer behaviour observed when the external stimuli are imposed by way of culture media. We continued to use the media from human-assisted reproduction as the test media and KSOM(aa) as the reference medium. Overall, when cell counts in the three germ layers were examined collectively for all twin pairs, statistical clustering analysis returned the correct pair associations in less than 10% of twin pairs (CSC medium, 3 of 35 pairs; GLOBAL medium, 0 of 36 pairs; GM501 medium, 0 of 33 pairs; G-TL medium, 0 of 36 pairs; SAGE 1-step medium, 1 of 36 pairs), resembling the situation in KSOM(aa) (Fig. 1E; Supplementary Table SII). We particularly examined the accumulation of EPI cells in individual embryos and the ratio between the EPI counts in twin and co-twin.

**Figure 2:**
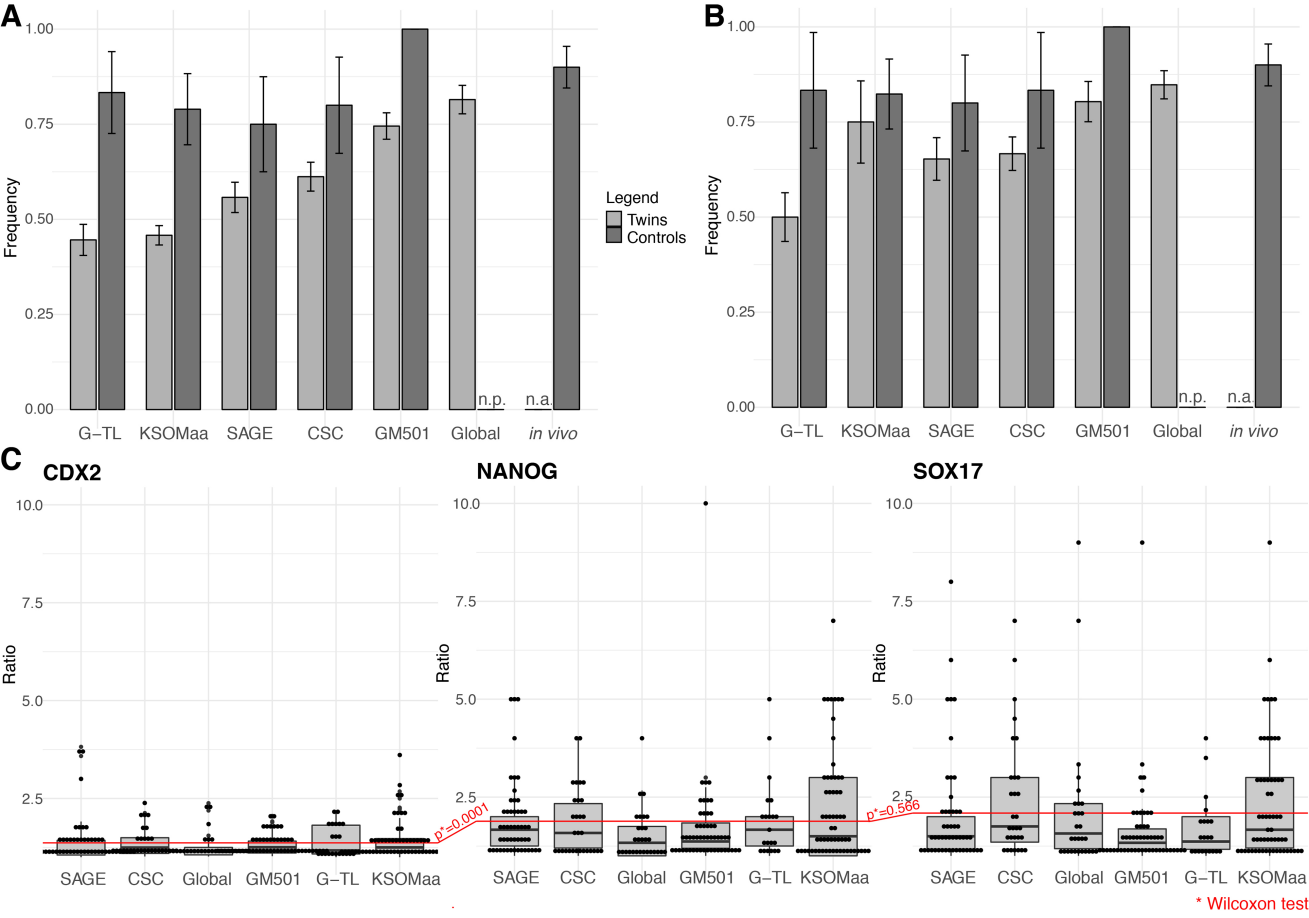
**The absolute number of EPI cells in MZ twin blastocysts varies, but the ratio twin/co-twin does not.** (**A**) Minority of the twin embryos cultured in KSOM(aa) accrued ≥4 EPI cells, compared to intact controls. While the proportion increased when the twins were cultured in one of the refined media (SAGE 1-step, GM501, G-TL, CSC, Global), the gap between twin embryos and intact controls persisted. Total starting numbers in (A): G-TL, 148 twins, 12 controls; KSOM(aa), 382 twins, 19 controls; SAGE, 156 twins, 12 controls; CSC, 165 twins, 10 controls; GM501, 157 twins, 17 controls; Global, 108 twins; *in vivo*, 30 controls. (**B**) Same as (A), except that only blastocysts with at least 50 cells were considered. Total starting numbers: G-TL, 62 twins, 6 controls; KSOM(aa), 16 twins, 17 controls; SAGE, 72 twins, 10 controls; CSC, 117 twins, 6 controls; GM501, 56 twins, 15 controls; Global, 92 twins; *in vivo*, 30 controls. (**C**) The ratio obtained from a higher cell count divided by the lower count (*C*^high^/*C*^low^), for each twin pair in each germ layer shows that the EPI imbalance persists no matter the cues from the culture environment. Abbreviations: n.p., not performed; n.a., not applicable; CDX2, trophectoderm; SOX17, primitive endoderm; NANOG, EPI. Dots in (C) are embryo pairs.

Only a minority of the twin blastocysts cultured in KSOM(aa) accumulated ≥4 EPI cells (Fig. 2A), which is considered to be a prerequisite for successful post-blastocyst development in mice (Morris *et al.*, 2012). Because the value of ≥4 EPI cells is regarded as critical in absolute terms (Morris *et al.*, 2012), the results are normalized to the number of blastocysts and not to the number of cells in the blastocysts. The minority proportion of twin blastocysts containing ≥4 EPI cells increased overall in SAGE 1-step, GM 501, G-TL, CSC and Global medium, without closing the gap relative to the intact controls (Fig. 2A). We also examined the blastocysts with at least 50 cells (Fig. 2B) since the total number of cells is lower in KSOM(aa) than in the media from human-assisted reproduction, and confirmed the previous finding (Fig. 2A). Thus, the lower proportion of blastocysts with ≥4 EPI cells is a conserved trait among twins. It is noteworthy that intact controls featured cases of an insufficient (<4) EPI cell number, prompting us to interrogate an *in vivo* group. Even when blastocysts were flushed from the uterus and thus, had no background of IVC, cases of EPI counts below the threshold of four were still recorded (Fig. 2A and B).

**Figure 3:**
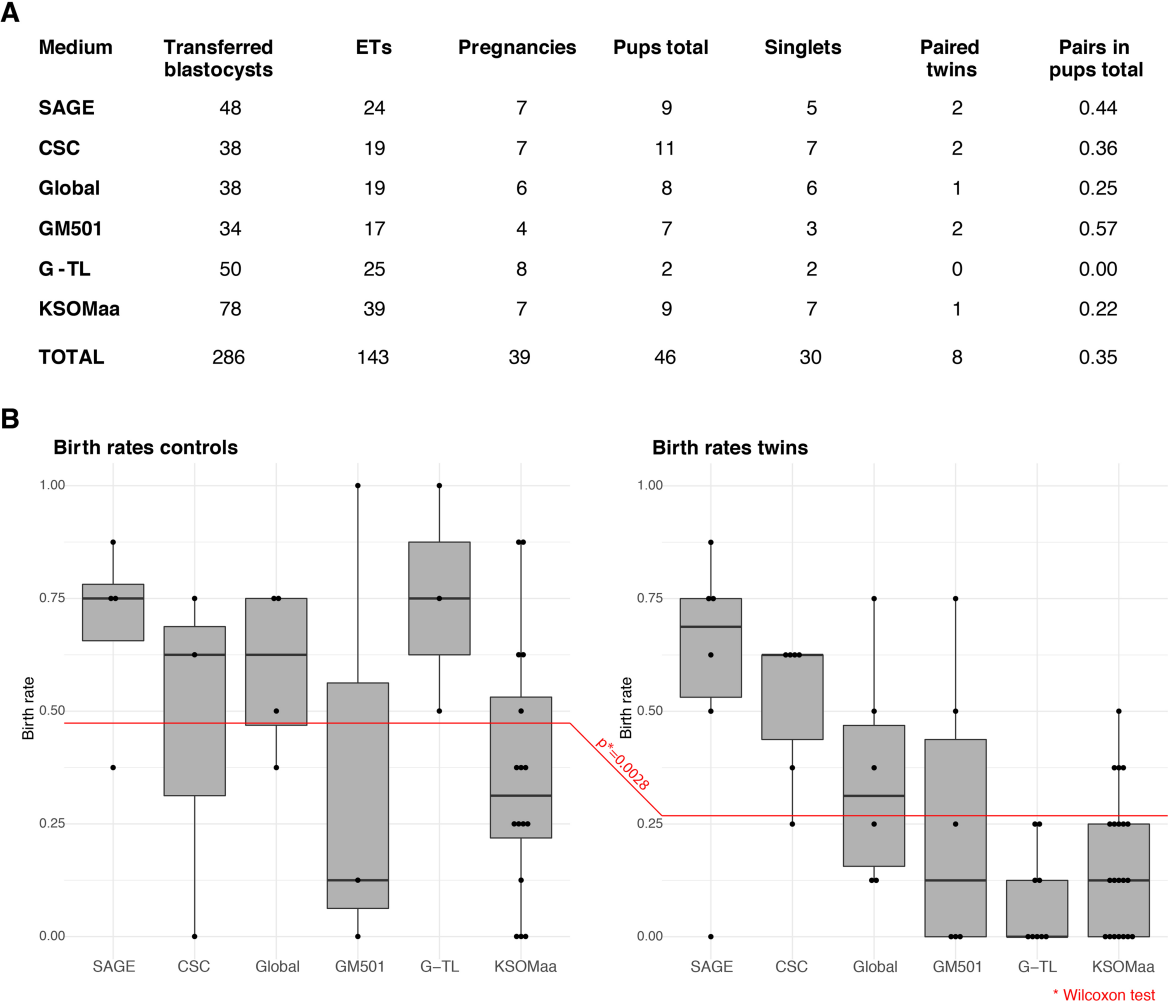
**EPI imbalance of MZ twin blastocysts does not recover after embryo transfer (ET) to the uterus.** (**A**) Embryo transfer of single pairs of MZ twins to the uterus (*n* = 2 in 1 female) produces single pups more frequently than pairs of pups. (**B**) Embryo transfer of pools of MZ twins to the uterus (*n* = 8 in 1 female) fares below transfer of control (non-manipulated) embryos. Points in the box plots are the birth rates per single ET (at least three replicates per medium). Total numbers of pups from ET of pools: SAGE 1-step, *n* = 28; GM 501, *n* = 2; G-TL, *n* = 6; CSC, *n* = 25; Global, *n* = 17; KSOM(aa), *n* = 28.

When the ratio between the EPI counts (NANOG) in twin and co-twin was examined, an imbalance was observed. We used the arithmetic ratio obtained from the higher count divided by the lower count (*C*^high^/*C*^low^) for each twin pair. Thus, balance and imbalance are easily identified from ratios near 1 or well above 1, respectively. An EPI imbalance (ratio ≈ 2) is extant, regardless of the culture environment (Fig. 2C). Unsurprisingly, this is also the case for primitive endoderm (SOX17), whose formation depends on the FGF4 produced by the EPI (Krawchuk *et al.*, 2013). In contrast to the EPI, the *C*^high^/*C*^low^ ratios for trophectoderm (CDX2) are not only similar across media but also near 1 (Fig. 2C), consistent with the notion that the imbalance affects the second cell lineage decision. We performed 143 transfers of two MZ blastocysts (twin and co-twin) per recipient, ensuring that each pair was transferred into the same uterus (using unfertilized oocytes as carriers), to test the possibility that EPI imbalance might simply be transient and would recover later. The birth of singlets exceeded the birth of pairs (Fig. 3A), regardless of the diverse culture media that produced heterogeneous blastocyst qualities (e.g. transcriptome, proportion of blastocysts with ≥4 EPI cells). A total of 16 out of 46 pups retrieved on E17.5 belonged to 8 complete pairs, while the remaining 30 pups were singlets (i.e. in 30 pairs, one member was missing). All these pups were outwardly normal and were not examined further. The 8:30 allocation observed to complete and incomplete pairs, respectively, is statistically different from the 38:0 allocation which would be expected if all MZ pairs proved to be totipotent in both members (Fisher’s exact test, *P* = 0.00001). We also repeated the embryo transfers using pools larger than two to allay the critique that transfers of two embryos per recipient are limiting in a litter-bearing species such as the mouse. We transferred groups of eight blastocysts to the uterus (83 transfers), followed by caesarean section on E17.5. While the probability of gestation is, of course, higher for transfers of eight embryos compared to two, the gap between the birth rates of half and intact embryos persisted (Fig. 3B). Thus, the biologically more rigorous but statistically weaker analysis of birth rates of single pairs documents that the intra-pair imbalance of totipotency occurs in 79% (30/38) of the pairs, despite the maximum extent of IVC support. The biologically less rigorous but statistically stronger analysis of the pools corroborates the findings obtained from the single pairs.

Altogether, these data show that the absolute number of EPI cells can vary in MZ half-embryos, but the imbalanced EPI ratio in twin pairs is invariant, irrespective of the culture environment. This *in vitro* behaviour reflects the birth rates *in vivo*. It is implausible that six different culture media produced the same effect. Therefore, our interim conclusion is that the persisting EPI imbalance is rooted not in regulative processes that are dependent on extrinsic factors (insofar as these processes can be modulated by culture media) but is rooted in intrinsic differences in developmental potential between blastomeres.

### Parthenogenesis reveals that the EPI formation imbalance is latent in oocytes

There is at least one confounder that makes imperfect EPI regulation complex to study in twins derived from zygotic embryos: the variability associated with the sperm entry point on the oolemma, which can be transmitted to one or the other blastomere; thus, making them differ from each other even before our experimental interventions start. If the sperm entry point is a primer of the EPI imbalance, then the imbalance should be less pronounced or absent in embryos formed exclusively by oocytes (parthenogenesis). In fact, the imbalance might be even easier to detect, since parthenogenetic embryos are more uniform owing to the synchronous chemical activation.

**Figure 4:**
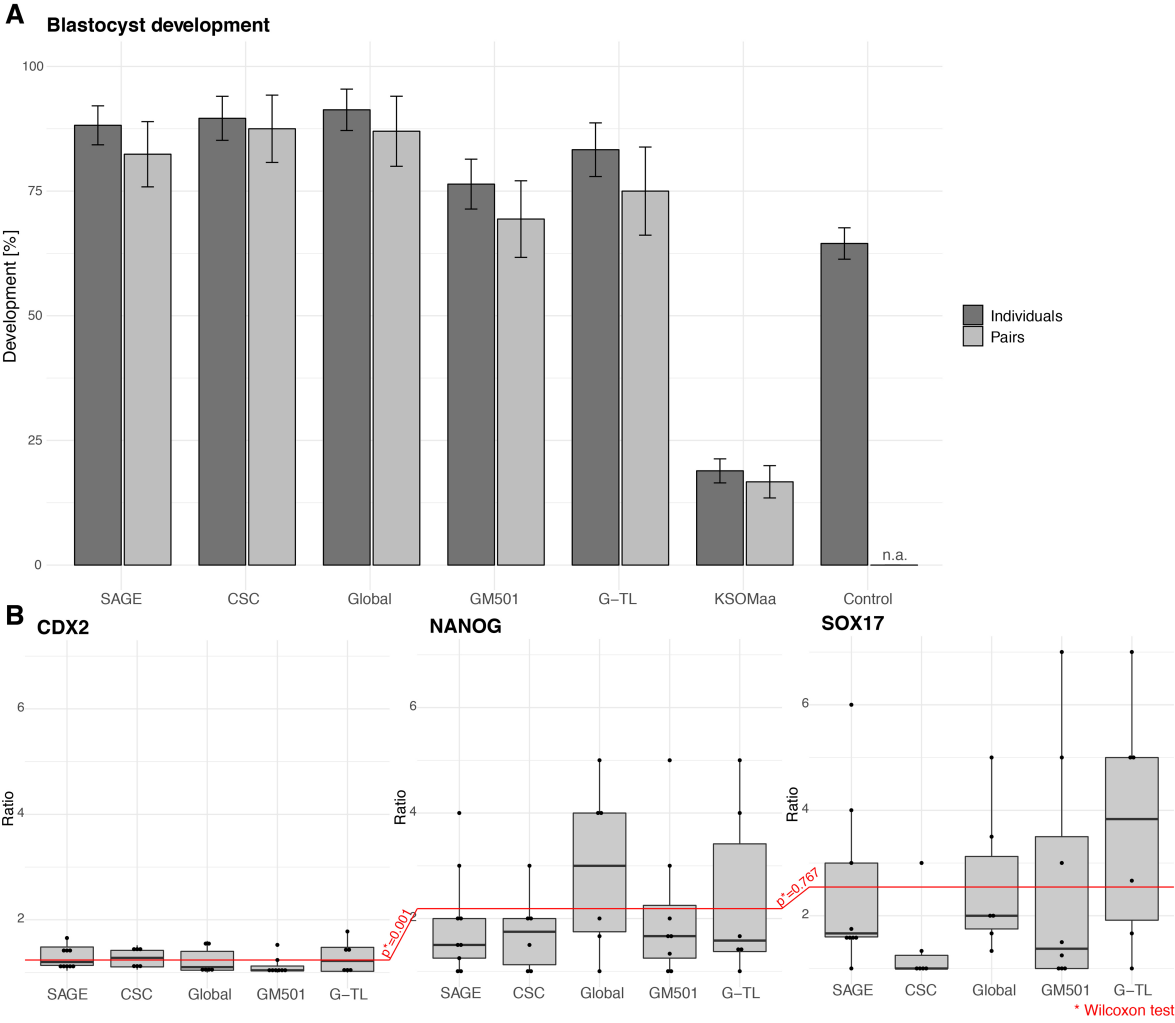
**EPI formation imbalance is latent in twin embryos from oocytes subjected to parthenogenetic activation.** (**A**) In the course of 281 bisections, we found that both blastomeres survived in 273 cases (97.3 ± 1.9%) and one blastomere survived in the remaining cases. Development to blastocyst was substantial, except in KSOM(aa) medium. Total starting numbers of parthenogenetic twins in A: SAGE, 34 pairs; CSC, 24 pairs; Global, 23 pairs; GM501, 36 pairs; G-TL, 24 pairs; KSOM(aa), 132 pairs; control, 231 non-bisected parthenogenetic embryos in KSOM(aa). (**B**) A total of 35 of the blastomere pairs that developed into pairs of blastocysts were subjected to cell lineage analysis (SAGE, *n* = 9; GM501, *n* = 8; Global, *n* = 6; G-TL, *n* = 6; CSC, *n* = 6). The ratio obtained from the higher count divided by the lower count (*C*^high^/*C*^low^) for each twin pair in each primary germ layer shows that the EPI imbalance persists no matter the cues from the culture environment. The erratic blastocyst formation of parthenogenetic twins in KSOM(aa) did not allow an examination of the effect of this medium on the primary germ layer composition. Abbreviations: CDX2, trophectoderm; SOX17, primitive endoderm; NANOG, EPI. Dots in (B) are embryo pairs.

Twins were derived efficiently from diploid parthenogenetic 2-cell stage embryos (B6C3F1) generated according to our established protocols for oocyte activation (Materials and Methods). After efficient 2-cell stage bisection, we treated the parthenogenetic pairs (zona-free) with the same media used previously to culture the MZ twins. Separated blastomeres were able to thrive in SAGE 1-step, GM501, G-TL, CSC or Global medium, while they were not able to thrive in standard medium KSOM(aa), in contrast to non-bisected parthenotes (Fig. 4A). Therefore, the outcomes of twinning after parthenogenesis are similar and yet distinguishable from those of twinning after fertilization. The cause of the poor development of parthenogenetic twins when cultured in KSOM(aa) transcends the scope of this study, but it underscores the importance of performing observations in multiple developmental environments. 

We applied the same pipeline used for the zygotic twin blastocysts to the parthenogenetic twin blastocysts to delve into the EPI imbalance. We analyzed the germ layer composition of blastocysts using the complete pairs, keeping track of the original pair associations (twin and co-twin). Across the culture media, the EPI counts (NANOG) in one member of the pair did not correlate with the EPI counts in the other member, measured by the ratio obtained from the higher count divided by the lower count (*C*^high^/*C*^low^), for each twin pair (Fig. 4B). This also applies to the primitive endoderm (SOX17), as has already been noted for the zygotic embryos, whereas the trophectoderms (CDX2) are in fair reciprocal concordance (*C*^high^/*C*^low^ ≈ 1; Fig. 4B).

We were interested in the consequences of EPI imbalance, but parthenogenesis impedes postimplantation development, making embryo transfer an impractical thing to do. Therefore, we adapted a recently established IVC system in which embryos can reach a stage *in vitro* that approximates to the egg cylinder stage *in vivo* (E5.5) (Bedzhov *et al.*, 2014). Parthenogenetic twin blastocysts were cultured for 3 days in IVC1 medium followed by IVC2 medium in 96-well plates and scored for the formation of mature structures resembling early egg cylinders (Supplementary Fig. S1). A total of 43% of the parthenogenetic pairs formed mature structures in both members (pair concordance), compared to 77% of dizygotic pairs. Conversely, a mature structure in one member and an immature structure in the other member (pair discordance) was observed in 47% of parthenogenetic pairs, compared to 20% of dizygotic pairs (total *N* = 77 and 44 pairs, respectively).

Altogether, the data presented so far show that the EPI imbalance (i) is independent of fertilization and (ii) does not recover in parthenogenetic embryos either until or after the blastocyst stage. Thus, these data raised the possibility that a maternal factor may play a role in determining the different developmental potencies of 2-cell blastomeres.

### Evidence of transcript patterning in parthenogenetic 2-cell embryos provides molecular correlates of the EPI formation imbalance

Prompted by the conserved discrepancy of EPI formation in most pairs of twins, we were interested to see whether there is any evidence of spatially localized developmental information that is relevant for the EPI and that differs in the sister blastomeres. This information could be carried by any kind of molecules. Findings of cellular mRNA gradients in lower species (Swalla and Jeffery, 1995; King *et al.*, 2005) suggest that if the interblastomere difference was also mediated by mRNA in mice, then we should be able to see it by super-resolution mRNA FISH (Raj *et al.*, 2008). Which mRNAs are going to exhibit spatial patterns was not known *a priori*, therefore, we first interrogated the transcriptome of individual 2-cell stage blastomeres to select those candidates that (i) exhibit more blastomere-to-blastomere variability of expression at the 2-cell stage and (ii) are part of the regulatory network of the EPI: *Cops3*, *Esrrb*, *Foxd3, Gbx2, Klf2*, *Klf4*, *Nanog*, *Pou5f1*/*Oct4*, *Sall4*, *Sox2, Tdgf1*/*Cripto* and *Tfcp2l1* (listed in alphabetical order; Hanna *et al.*, 2002; Yan *et al.*, 2003; Dunn *et al.*, 2014; Boroviak *et al.*, 2015; Fiorenzano *et al.*, 2016).

**Figure 5:**
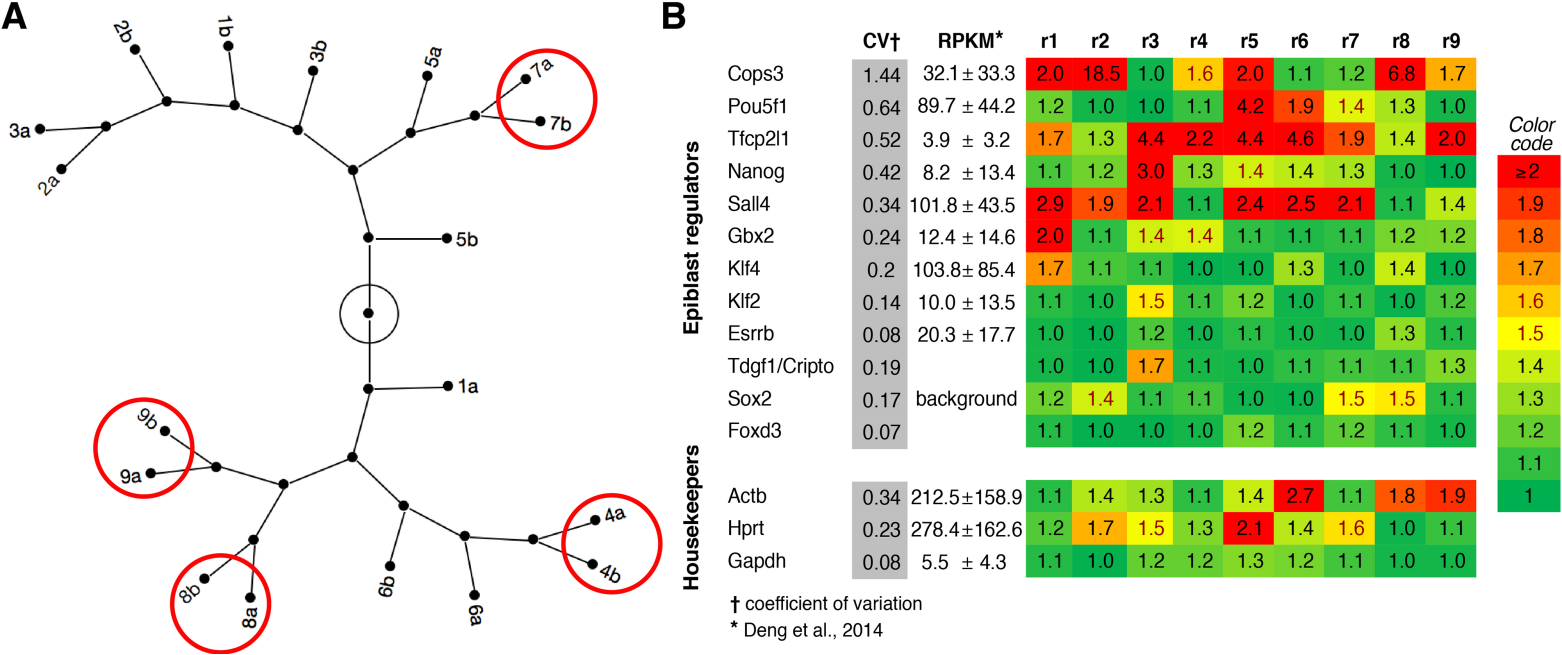
**EPI formation imbalance in blastocysts correlates with differences of EPI-associated gene expression at the 2-cell stage.** (**A**) Transcriptome analysis (Affymetrix) of blastomere pairs from nine parthenogenetic 2-cell embryos produced in KSOM(aa). Constellation maps of the transcriptomes of individual blastomeres identified with both a number (for the original embryo subjected to bisection) and a letter (for the two members resulting from bisection). The transcriptomes were subjected to hierarchical clustering analysis to see whether they would return the original pair associations (e.g. 7a–7b), which they did in a minority of cases. (**B**) Transcriptomes were queried for mRNAs of EPI-related genes. The heat map shows the mRNA interblastomere ratios of EPI-related and housekeeping genes, ranked by coefficient of variation †. *mRNA expression level (RPKM) at the 2-cell stage is derived from Deng *et al* (2014). It should be noted that the embryos analyzed by Deng *et al*. were produced by fertilization. RPKM, reads per kilobase of transcript per million mapped reads.

The transcriptomes of nine pairs of parthenogenetic blastomeres were matched correctly with each other in only four pairs (Fig. 5A), as determined by hierarchical cluster analysis of the transcriptomes (deposited dataset GSE111589; Supplementary Table SIII). This corroborates the view that the first cleavage can produce both balanced and imbalanced blastomeres, regardless of sperm-associated processes. We used the ratio of each mRNA expression level between the sister blastomeres, always dividing the higher value by the lower value, across the nine pairs to extract the blastomere-to-blastomere variability at the 2-cell stage. As expected, the interblastomere ratios of housekeeping mRNAs (Mamo *et al.*, 2007) were not significantly different from 1 (Fig. 5B). Some of the EPI-related mRNAs were detected at a background level with a low coefficient of variation between the blastomere pairs, while others were detected at a higher level with additionally a higher coefficient of variation. *Cops3,* with the highest coefficient of variation, stands out among the EPI-related genes tested, followed by *Oct4*/*Pou5f1* (Fig. 5B).

**Figure 6:**
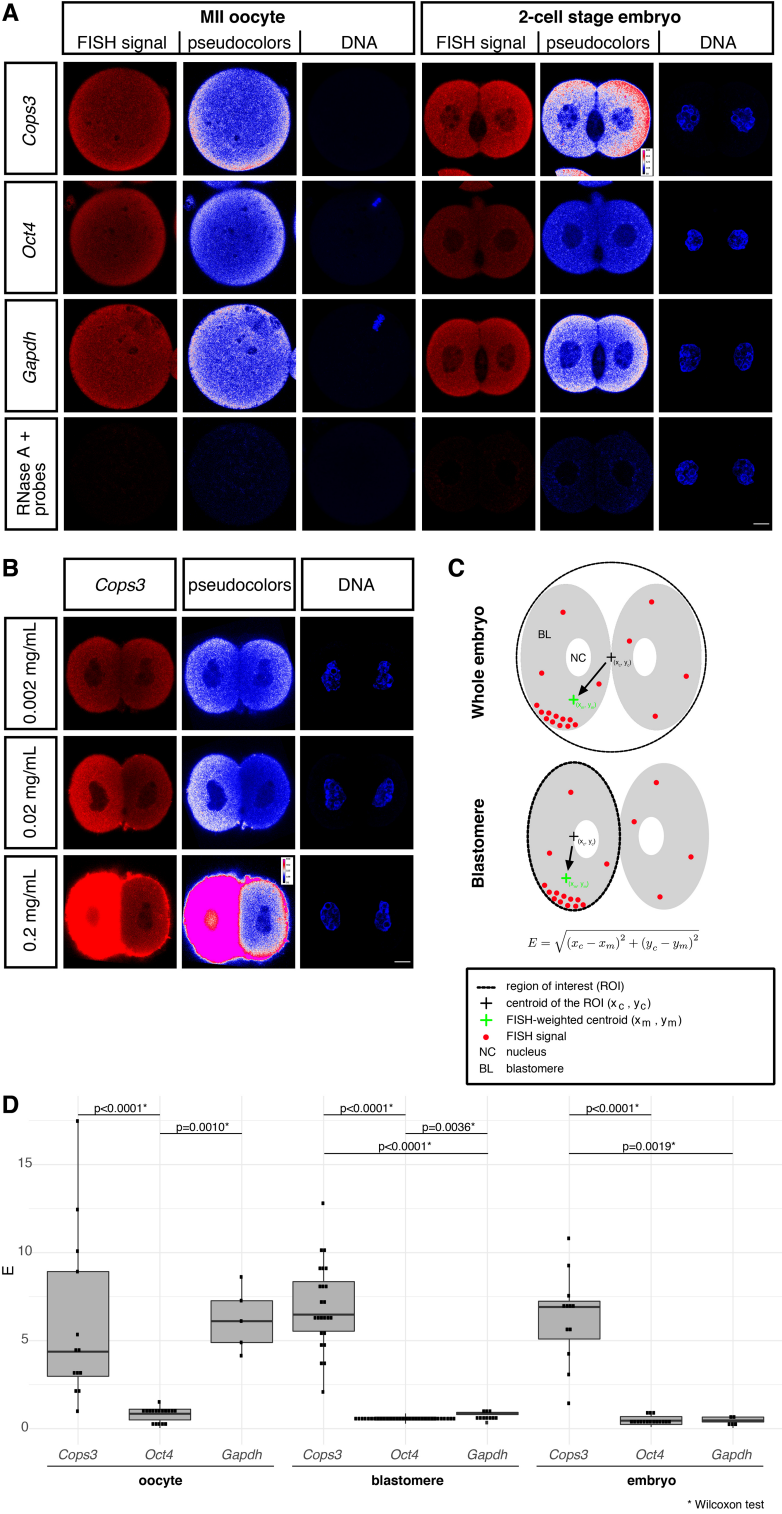
**Sub-embryo and sub-blastomere transcript patterning in parthenogenetic 2-cell embryos.** (**A**) Maximum intensity projections of the confocal sections captured in the equatorial region of oocytes and consistently oriented 2-cell embryos are shown, demonstrating the much higher uniformity of *Gapdh* mRNA FISH signal compared to *Cops3*. Note the complete absence of FISH signal when hybridization was conducted after treatment with RNase (probe for oocytes is *Cops3*, probe for 2-cell embryos is *Gapdh*). FISH signal from Quasar® 670 fluorochrome is shown after normalization using identical settings for contrast and brightness. Pseudocolours are used to emphasize the differences of mRNA FISH signal distribution. DNA was stained with DAPI. (**B**) Same as in (A), except that one blastomere was injected with 0.002, 0.02 or 0.2 mg/mL *Cops3* mRNA to further demonstrate the specificity of the FISH signal. Size bar, 10 μm. (**C**) Degree of uniformity of FISH signal was captured by the mathematical measure of eccentricity (abbreviated as ‘E’). (**D**) There is substantial eccentricity of *Cops3* but not of the Gapdh signal in embryos and their blastomeres, attesting to the genuine inhomogeneity of the maternal cytoplasm in terms of *COP9 signalosome complex subunit 3* (*Cops3*) mRNA. Dots stand for individual metaphase II (MII) oocytes, individual blastomeres, individual embryos. Numbers of MII oocytes examined: *Cops3*, *n* = 13; *Gapdh*, *n* = 5; *Oct4*, *n* = 17. Numbers of 2-cell embryos examined: *Cops3*, *n* = 12; *Gapdh*, *n* = 5; *Oct4*, *n* = 16. Eccentricity values were compared using the Wilcoxon test.

We used mRNA FISH in super-resolution mode confocal microscopy to assess the spatial pattern of *Cops3* in parthenogenetic 2-cell embryos. In addition to *Cops3*, we also inspected *Gapdh* as a housekeeping control, and *Oct4*/*Pou5f1* as the second most differently expressed mRNA after *Cops3* (Fig. 5B). Precursor MII oocytes were also examined to see whether the spatial mRNA patterns observed at the 2-cell stage were related to patterns from a previous stage (Fig. 6A). Each molecule of the target mRNA in super-resolution FISH is bound by 48 oligonucleotides targeting the whole mRNA length, and each oligonucleotide is tagged with a fluorophore (Stellaris® probes).

We applied the following three controls to show that our imaging protocol did not produce obvious artefacts. Firstly, pre-treatment of the specimens with RNase A eliminated the fluorescent signal, thus, confirming its RNA origin in the rest of the experiments (Fig. 6A). As a second control, rotating the specimen by 90° on the stage of the microscope resulted in a rotation of the *Cops3* pattern (Supplementary Fig. S2); thereby, excluding a microscope’s optical path artefact. As a third control, microinjection of exogenous *Cops3* mRNA into one of the two blastomeres followed by fixation 10 min later resulted in a stronger FISH signal in the injected blastomere (Fig. 6B); thus, confirming the specific *Cops3* origin of the signal. Imaging was conducted only on those 2-cell embryos that were lying on a dish with both nuclei on the same focal plane to facilitate their observations. Confocal sections were taken in the largest region (2.2 μm across the cell’s equator) of these controls and in the experiments to follow. This confinement is necessary to allay the concerns about the length of the light path and bleaching of signal during laser scanning, which is more problematic in the Z dimension.

**Figure 7:**
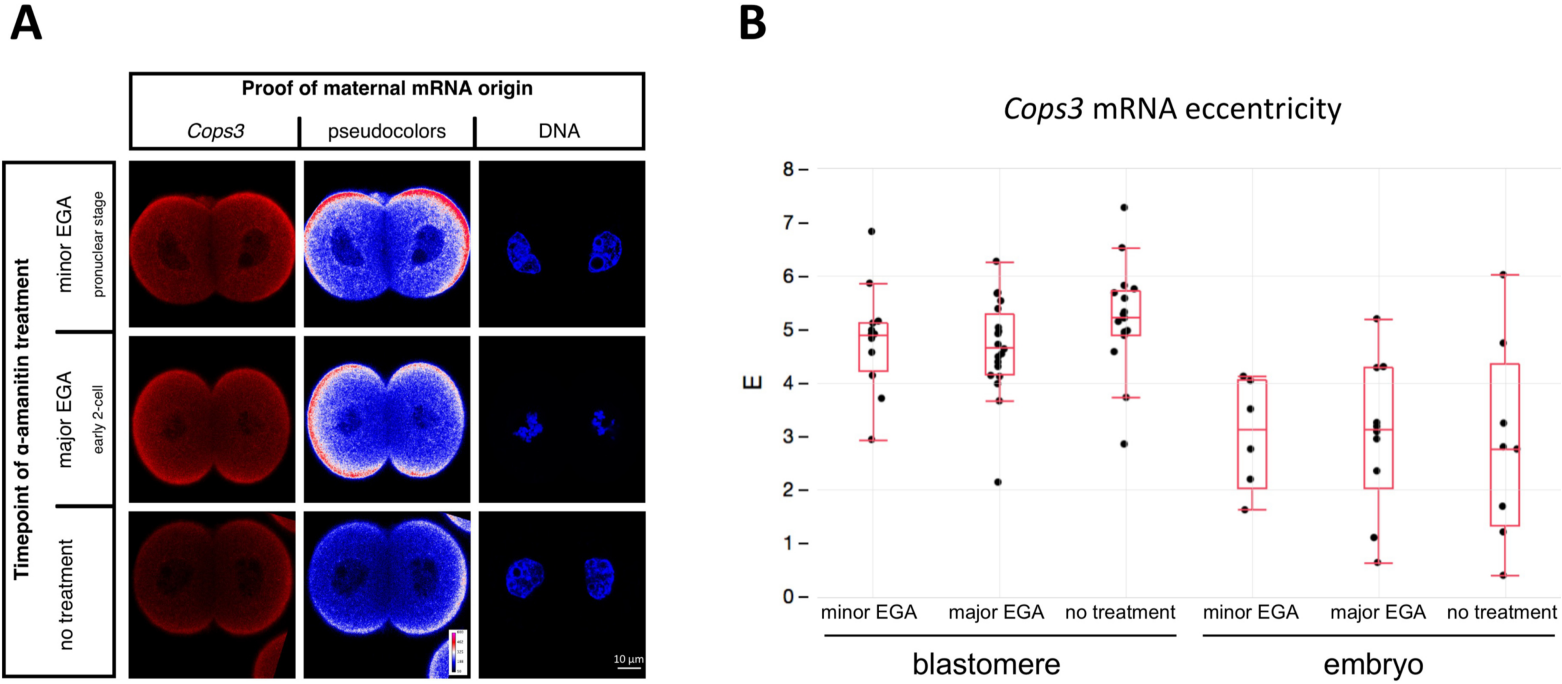
***Cops3* mRNA spatial distribution is independent of *de novo* transcription.** (**A**) Maximum intensity projections of the confocal sections captured in the equatorial region of consistently oriented 2-cell embryos are shown, demonstrating very similar mRNA FISH signal distribution irrespective of treatment with α-amanitin. FISH signal from Quasar® 670 fluorochrome is shown after normalization using identical settings for contrast and brightness. Pseudocolours are used to emphasize the anisotropy of mRNA FISH signal distribution. DNA was stained with DAPI. (**B**) Eccentricity of *Cops3* signal is not significantly (Wilcoxon test) different within the blastomere and embryo groups. Dots stand for individual blastomeres or individual embryos. Numbers of blastomeres (embryos) examined: minor EGA, *n* = 12 (6); major EGA, *n* = 20 (10); no treatment, *n* = 16 (8). Abbreviations: EGA, embryonic genome activation.

Analysis of maximum intensity projection images revealed a subtle but reproducible transcript pattern of *Cops3* mRNA in the X-Y dimension, both within and between the parthenogenetic blastomeres, in contrast to the more uniform X-Y signal of *Gapdh* and *Oct4* mRNAs. This pattern was not unique to parthenogenetic 2-cell embryos but was also observed in fertilized counterparts. We borrowed the concept of eccentricity of the FISH signal (Fig. 6C) to describe the transcript pattern in systematic terms for quantitative analysis (Park *et al.*, 2012). Eccentricity is defined as the linear distance between the geometrical centre of the image and its intensity-weighed centre of mass (see Materials and Methods). Accordingly, the more the distribution of an mRNA is spatially inhomogeneous, the higher the eccentricity (except perhaps when the inhomogeneity is radial). The images used for this analysis were kept in the native red channel and normalized using identical settings for contrast and brightness, contrary to the pseudocoloured images, which are intended for demonstration purposes only. The analysis of 2-cell embryos hybridized for *Cops3*, *Gapdh* and *Oct4* revealed a significantly higher eccentricity of *Cops3* in both blastomeres and whole embryos (Fig. 6D; additional embryos are shown in Supplementary Fig. S3). To clarify the biological origin of eccentricity, pronuclear oocytes as well as early 2-cell embryos were cultured in the presence of RNA polymerase inhibitor α-amanitin until the late 2-cell stage, when they were processed for mRNA FISH analysis together with untreated 2-cell embryos and MII oocytes. A subset of the treated embryos were kept in culture but failed to reach the 4-cell stage, thereby proving the α-amanitin effect. Since inhomogeneous FISH signal distribution was already present in MII oocytes (Fig. 6A and D) and FISH signal eccentricity was still present after α-amanitin treatment (Fig. 7), the results imply that the spatial distribution of *Cops3* mRNA depends not on embryonic genome activation (EGA) but on maternally supplied factors. This is also consistent with a transcriptome profiling showing that *Cops3* mRNA declines from MII oocytes to 1-cell and early 2-cell stage embryos, followed by a steady increase from the late 2-cell stage onwards (Supplementary Fig. S4; data extracted from deposited dataset GSE110599).

Altogether, the data above show the blastomere-to-blastomere variability of EPI regulators and reveal both the sub-embryo and subcellular differential localization of one (*Cops3*) but not another (*Oct4*) of the EPI regulators *in situ*. Sister blastomeres are clearly not mirror images of one another but have a subtle transcriptomic patterning, which is not α-amanitin-sensitive, hence of maternal origin. In conjunction with the developmental rates, these data converge on the hypothesis that the transmission of factors that are relevant for totipotency from zygote to 2-cell stage blastomere has, at least to some extent, maintained some aspects of segregation. These findings provide a reason to discuss to what extent they may be in line with the ‘ooplasmic segregation hypotheses’ that were formulated in the 1950s through to the 1980s (Tarkowski, 1959; Seidel, 1960; Denker, 1976; Gärtner, 1990).

## Discussion

Early mammalian development is generally viewed as regulative, flexible and responsive to external perturbation, including experimental intervention. While multiple investigators have recognized a bias or preference in the differentiation of the first two blastomeres towards the embryonic and abembryonic part of the mouse embryo (Gardner and Davies, 2003; Motosugi *et al.*, 2005; Bischoff *et al.*, 2008), nobody has questioned whether both blastomeres were still totipotent. In this study, we documented interblastomere differences of developmental potential (ability to form EPI) that evade the regulative capacity of the embryo. This is an observation that cannot be easily explained without positing some role for the oocyte-borne factors. These considerations led us to investigate the transmission of totipotency from zygote to 2-cell stage blastomere. We propose a degree of spatial organization of oocyte cytoplasmic factors that have developmental significance and which, at least in a proportion of cases, are transmitted differentially to the first two blastomeres. This proposal is backed by three lines of evidence: (i) functional evidence (EPI lineage contribution and EPI-related *Cops3* mRNA expression differs between sister blastomeres); (ii) temporal evidence (inhomogeneity of *Cops3* mRNA spatial distribution is present already in precursor MII oocytes) and more importantly, (iii) direct *in situ* evidence (inhomogeneity of *Cops3* mRNA spatial distribution persists when EGA is prevented).

The idea that sister 2-cell blastomeres should be equally potent was based on the possible success of obtaining experimental twins after the blastomeres had been separated from each other, although this success was only relatively rare. One can generally never exclude the possibility that the mechanisms operating following experimental intervention are different from those that operate during normal undisturbed development. The progenies of the first two blastomeres compete for space within the zona pellucida in normal development, for example, and the first-to-cleave blastomere has an edge over the other blastomere in populating the inner part of the morula/blastocyst. Therefore, when the two blastomeres are separated, they should enjoy same degrees of freedom; however, the separation is invasive. Even though the separation of 2-cell blastomeres is a mild action, compared to actions taken in other studies (poking, Tarkowski, 1959; piezoelectric scrambling, Evsikov *et al.*, 1994; ultracentrifugation, Mulnard and Puissant, 1984; cytoplasm aspiration, Zernicka-Goetz, 1998; Ciemerych *et al.*, 2000), we felt it was important to confirm that the separation *per se* was not causing the EPI imbalance. This was shown (among various controls) by the accumulation of very similar numbers of cells in the two twins if they are added together compared to non-manipulated blastocysts. This similarity is in accord with our previous finding that gene expression differences between half-blastocysts and non-manipulated controls are spurious (Casser *et al.*, 2017). In contrast to the negligible effect of the bisection, the perturbation introduced by culture media featured broad changes in the transcriptome and also increased the otherwise low proportion of half-blastocysts containing enough EPI cells to theoretically support postimplantation development (Morris *et al.*, 2012). The number of EPI cells had been increased previously via pharmacological agents (small molecule inhibitors of the mitogen-activated protein kinase and glycogen synthesis kinase 3, known as ‘2i’; Morris *et al.*, 2012). This was not an option in our study, because our intention was to use full development as an endpoint (2i treatment works via the interconversion of EPI and primitive endoderm, depleting the latter, and this depletion would impede full development.) We relinquished the pharmacological agents in favour of the culture media. Although MZ half-embryos thrived under superior embryo culture conditions and more of them progressed to birth than usual, the singletons persisted and continued to be as frequent as the complete pairs, in spite of the identical uterine environment. Thus, the EPI imbalance that affects the twin blastocysts does not recover afterwards. Conserved EPI imbalance was also observed in parthenogenetic 2-cell embryos, in contrast to a previous report that the first two parthenogenetic blastomeres have no tendency to follow different fates (Piotrowska and Zernicka-Goetz, 2002). Unfortunately, a comparison of these two studies does not contribute to the discussion, because the protocols of parthenogenetic activation were different and the blastocyst cells were scored by different criteria, i.e. position of the cells (Piotrowska and Zernicka-Goetz, 2002) versus molecular identity of the cells (this study).

The fact that the EPI imbalance was conserved under all tested culture conditions that release more embryonic regulation suggests that the imbalance is independent of the regulation itself. The companion observation, i.e. that an EPI imbalance is shared by zygotic and parthenogenetic embryos, suggests that the common ancestor of these embryos, namely the maternal cytoplasm, is likely to be the hinge point. Maternal cytoplasm supports various processes whose perturbation can be conducive to interblastomere differences, for example, errors of chromosome segregation and subcellular localization of gene products of any kind. Errors of chromosome segregation at the first embryonic division are unlikely to cause the EPI imbalance, because we showed that a large proportion of twin blastocysts were impaired in one member of the pair already at E5 (*in vitro* ‘postimplantation’ assay; Bedzhov *et al.*, 2014), whereas most of the aneuploid karyotypes do not cause mouse embryo demise until E7 (Gropp, 1982; Lightfoot *et al.*, 2006). If not aneuploidy, then the subcellular localization of gene products could cause interblastomere differences upon apportioning of the zygotic cytoplasm to the daughter cells. Various studies (Gardner, 1996, 1997; Piotrowska and Zernicka-Goetz, 2001, 2002; Fujimori *et al.*, 2003; Plusa *et al.*, 2005) have described a bias in the contribution of 2-cell stage blastomeres to the embryonic and abembryonic compartment of the blastocysts, although subsequently, the blastomere progenies in the foetuses contributed to both embryonic and extraembryonic tissues (Fujimori *et al.*, 2003; Li *et al.*, 2017). In contrast to these studies in which the blastomeres were kept together, our experimental design revealed different propensities of the blastomeres for full development when they were separated from each other. It should be possible to find molecular correlates; for instance, those that document how sister blastomeres are not mirror molecular images of one another. However, convincing *in situ* evidence of molecular patterning has been scarce so far and should it exist, its functional relevance has been viewed with scepticism since scrambling, aspiration or stratification of the cytoplasm had no obvious consequence for development (Mulnard and Puissant, 1984; Evsikov *et al.*, 1994; Zernicka-Goetz, 1998; Ciemerych *et al.*, 2000). However, this lack of effect can be questioned, because a cytoplasmic region positioned underneath the cell membrane (cortical) would be less influenced by these interventions. As to which specific gene candidates to pursue, the list of candidates is unsatisfactory at present. *Xenopus* studies teach us that specific RNA localization is common in oocytes of this organism (King *et al.*, 2005), while in mammals, specific RNA localization has not been reported, although it would be expected. For example, it would be expected from the non-uniform distribution of RNA-binding proteins MOEP19 (Herr *et al.*, 2008) and of the RNA-binding proteins hnRNPA1, eIF4A3, 4E-BP1 and CPEB4 (Jansova *et al.*, 2018), but apart from these indirect possibilities, the only report of an mRNA as such having a non-uniform cellular distribution is that of *Cdx2* in the 8-cell embryo (Skamagki *et al.*, 2013). Although maternal effect gene products are intriguing candidates, since they are required in development while they may be dispensable for other vital functions, there is currently no direct evidence implicating the zygotic asymmetry of these gene products in developmental cell lineage and axis determination (Condic, 2016). Additional candidates are not many, e.g. LEPTIN, STAT3, BAX, BCL-X, TGFβ2, VEGF, cKIT, EGF-R (Antczak and Van Blerkom, 1997, 1999), SNAI1 and SNAI2 (Bell and Watson, 2009), hCG (Edwards and Hansis, 2005) and GADD45a (Rosas *et al.*, 2018). Of note, some of these candidates appear to be present in oocytes or blastomeres in the form of, for example, polarized domains of proteins or mRNAs (Antczak and Van Blerkom, 1997). If, indeed, there is positionally localized molecular information laid down in the oocyte, then this information is apportioned unequally between blastomeres due to the variability in the orientation of the zygotic cleavage plane. Already in 1957, Albert Dalcq recognized, referring to mammals that ‘it must be made clear from the outset that there is nothing “determinate” about the process of cleavage’ (Dalcq, 1957). Clearly, suggestions for more candidates are overdue and the next logical step would be to search *in situ* for gene products that are differently abundant in the sister blastomeres.

We screened genes presenting more frequent interblastomere difference than others, focusing on those genes that have a role in EPI formation. Although totipotency and cell lineage formation is probably decided by more than one factor, transcriptome analysis directed our interest to one factor, *Cops3*. This encodes the third subunit of the COP9 signalosome protein complex, which was shown to be essential for EPI maintenance and proliferation in peri-implantation mouse embryos (Yan *et al.*, 2003) and is the most differently expressed among the EPI-related mRNAs that are detected at the 2-cell stage, directly followed by *Pou5f1* (*Oct4*). As we queried the expression of *Cops3 in situ* using Stellaris® probes in confocal imaging, we knew we had to be very cautious, because: (i) non-homogeneous signal distribution may be caused by the fixative (Littwin and Denker, 2011); (ii) extended focus in a spherical object is bound to create projection images with a gradient of fluorescence between the centre and periphery and (iii) differences in fluorescence intensity in the Z dimension can be due to bleaching. Therefore, we examined the sister blastomeres next to each other in the sole equatorial region (10 confocal slices corresponding to a total of 2.22 μm) to minimize these problems. Our image analysis in the X-Y dimension *in situ* revealed regions of blastomeres with a higher or lower intensity of *Cops3* mRNA FISH signal, whereas *Gapdh* mRNA was more uniformly distributed and *Oct4* mRNA was even more so. To the best of our knowledge, while non-uniform sub-blastomere distribution of an mRNA has been shown to be critical for the first cell lineage decision in the 8-cell stage mouse embryo (*Cdx2*; Skamagki *et al.*, 2013), non-uniform sub-blastomere distribution of other mRNAs at the 2-cell stage has not been reported until now.

A major open question is whether the differences in blastomere totipotency after the first cleavage are a fortuitous event or a developmentally programmed one, and whether differences are generated *de novo*. In teleological terms, we consider that there is no compelling reason for mammalian 2-cell embryos to maintain totipotency in both blastomeres, because (i) the sheltered environment of the mother, as provided in viviparous species, makes cell injury improbable and (ii) the natural tendency of increase in entropy is better satisfied when the sister blastomeres are not equal. The latter can be tolerated if the system provides enough regulative capacity via cell–cell interactions. Accordingly, it is tempting to consider that there has been no evolutionary pressure to maintain cellular totipotency throughout early cleavage and that there is no mechanism to ensure totipotency in both blastomeres. Therefore, either both or just one could be totipotent, fortuitously, depending, for instance, on how the randomly oriented plane of the first zygotic cleavage (Louvet-Vallee *et al.*, 2005; Zernicka-Goetz, 2005) apportions some spatially localized developmental information laid in the ooplasm. This possibility is supported by previous independent work (Antczak and Van Blerkom, 1999). As to the specific identity of this developmental information, we expect the products of multiple genes to be involved, and the case of *Cops3* does not mean that this is the quintessential gene to study for the next few years, but its case demonstrates that differences are not generated *de novo* through, e.g. embryonic genome activation (α-amanitin experiment). It should be interesting to test whether other gene products, in addition to *Cops3*, make a contribution to interblastomere differences at the 2-cell stage. Two long non-coding RNAs (*LincGET* and *Neat1*), for example, are differently expressed in the sister blastomeres of the same 2-cell embryo and expression levels correlate with the contribution of the blastomere progeny to the ICM (Hupalowska *et al.*, 2018; Wang *et al.*, 2018). Such differences between 2-cell blastomeres would probably not matter for the totipotency of the normal embryo of this stage, i.e. comprised of two blastomeres, since one blastomere would compensate for the deficit of the other (unless fragmentation would occur; Antczak and Van Blerkom, 1999), but it does matter if the blastomeres are separated. This scenario is reminiscent of the historical proposals that: (i) postnatal phenotypic differences in MZ twins continue to occur even when the environment is standardized, because they depend on ooplasmic influences, which are effective at or before fertilization (Gärtner, 1990) and (ii) there is an embryonic knot determinant in the ooplasm, which is required to form a pluripotent ICM, and how equally this determinant is apportioned to the sister blastomeres depends on the variability of the first cleavage plane orientation (Seidel, 1960).

## Supplementary Material

Casser_et_al_Supplementary_figure_1_Aug12_2019_gaz051

Casser_et_al_Supplementary_figure_2_Aug12_2019_gaz051

Casser_et_al_Supplementary_figure_3_Aug12_2019_gaz051

Casser_et_al_Supplementary_figure_4_Aug12_2019_gaz051

Casser_et_al_Supplementary_table_1_gaz051

Casser_et_al_Supplementary_table_2_gaz051

Casser_et_al_Supplementary_table_3_gaz051
