# Supplementary figures and images for: Differences in blastomere totipotency in 2-cell mouse embryos are a maternal trait mediated by asymmetric mRNA distribution
Source: Mol Hum Reprod. 2019 Sep 4;25(11):729–44. doi: 10.1093/molehr/gaz051 (PMC6884417; doi:10.1093/molehr/gaz051)

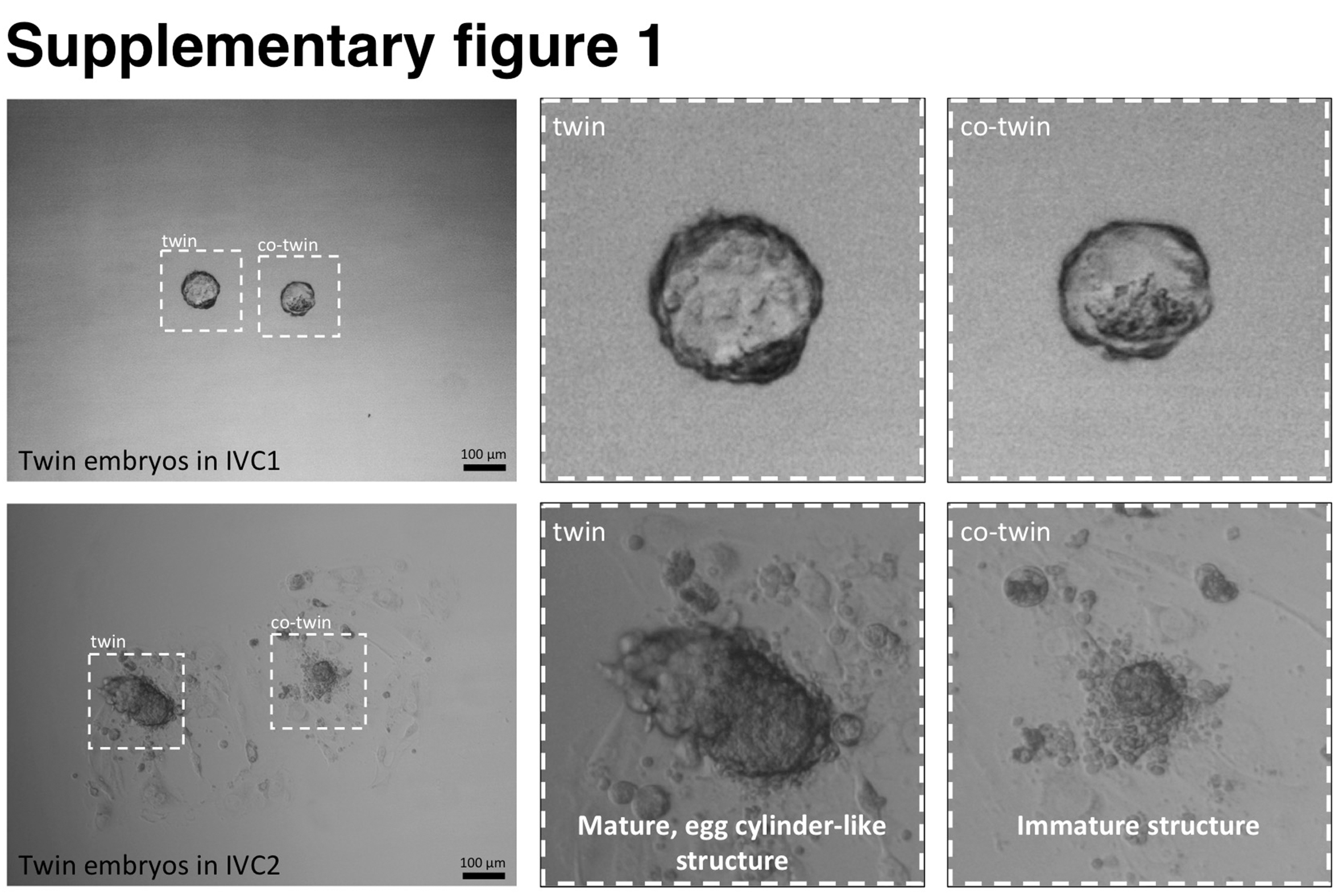

Supplement: Casser_et_al_Supplementary_figure_1_Aug12_2019_gaz051 [file casser_et_al_supplementary_figure_1_aug12_2019_gaz051.jpeg]

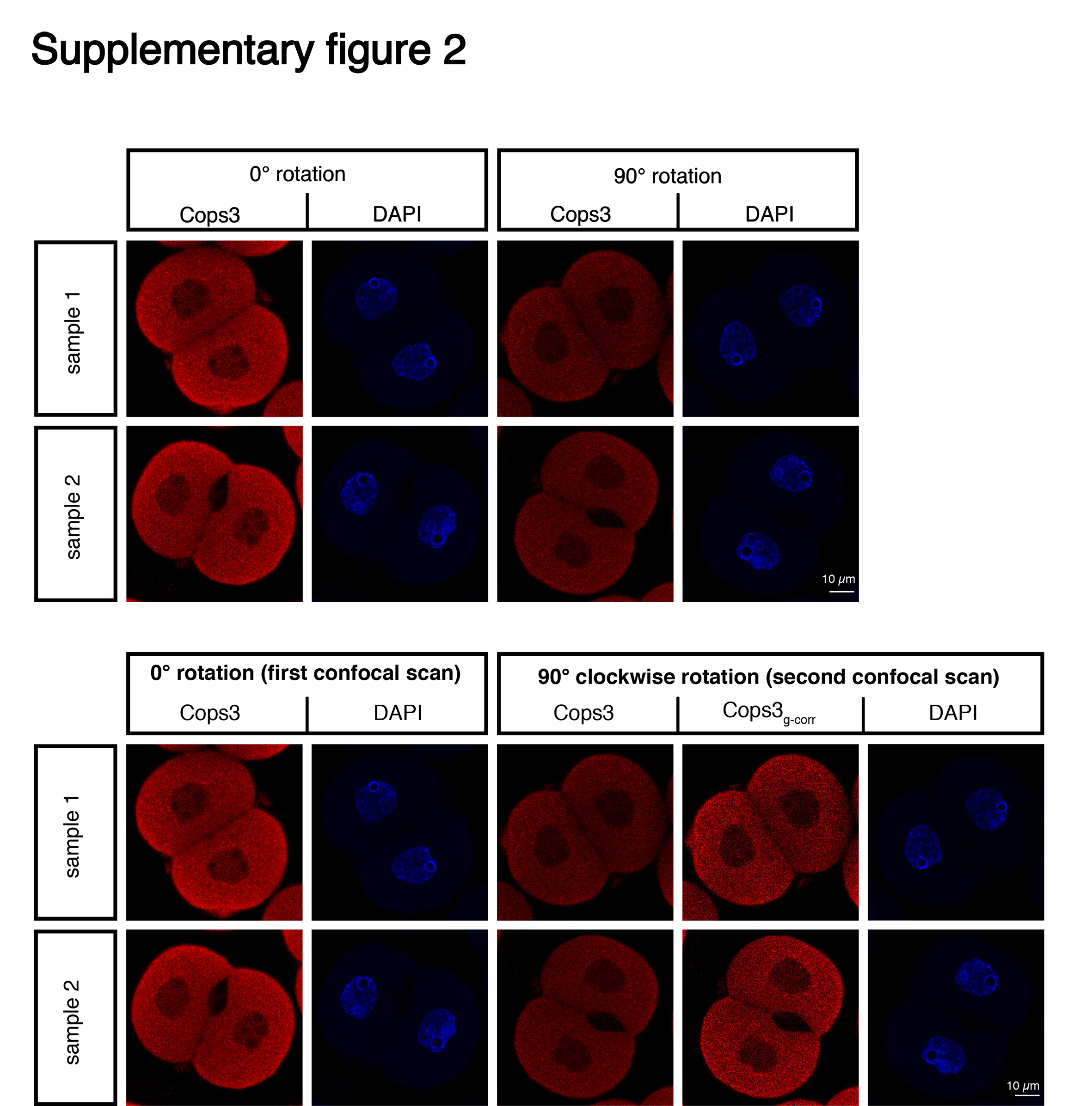

Supplement: Casser_et_al_Supplementary_figure_2_Aug12_2019_gaz051 [file casser_et_al_supplementary_figure_2_aug12_2019_gaz051.jpeg]

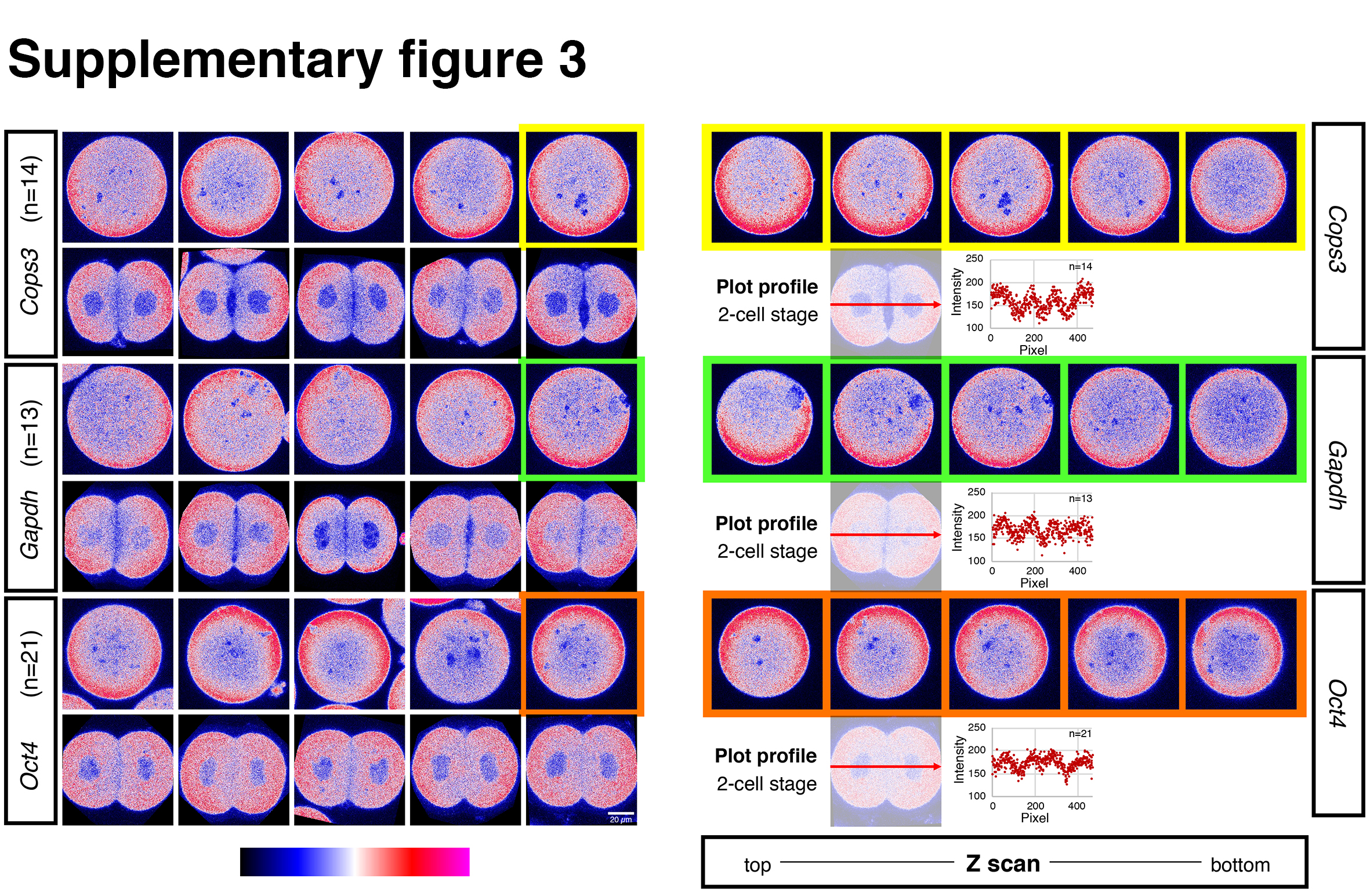

Supplement: Casser_et_al_Supplementary_figure_3_Aug12_2019_gaz051 [file casser_et_al_supplementary_figure_3_aug12_2019_gaz051.jpeg]

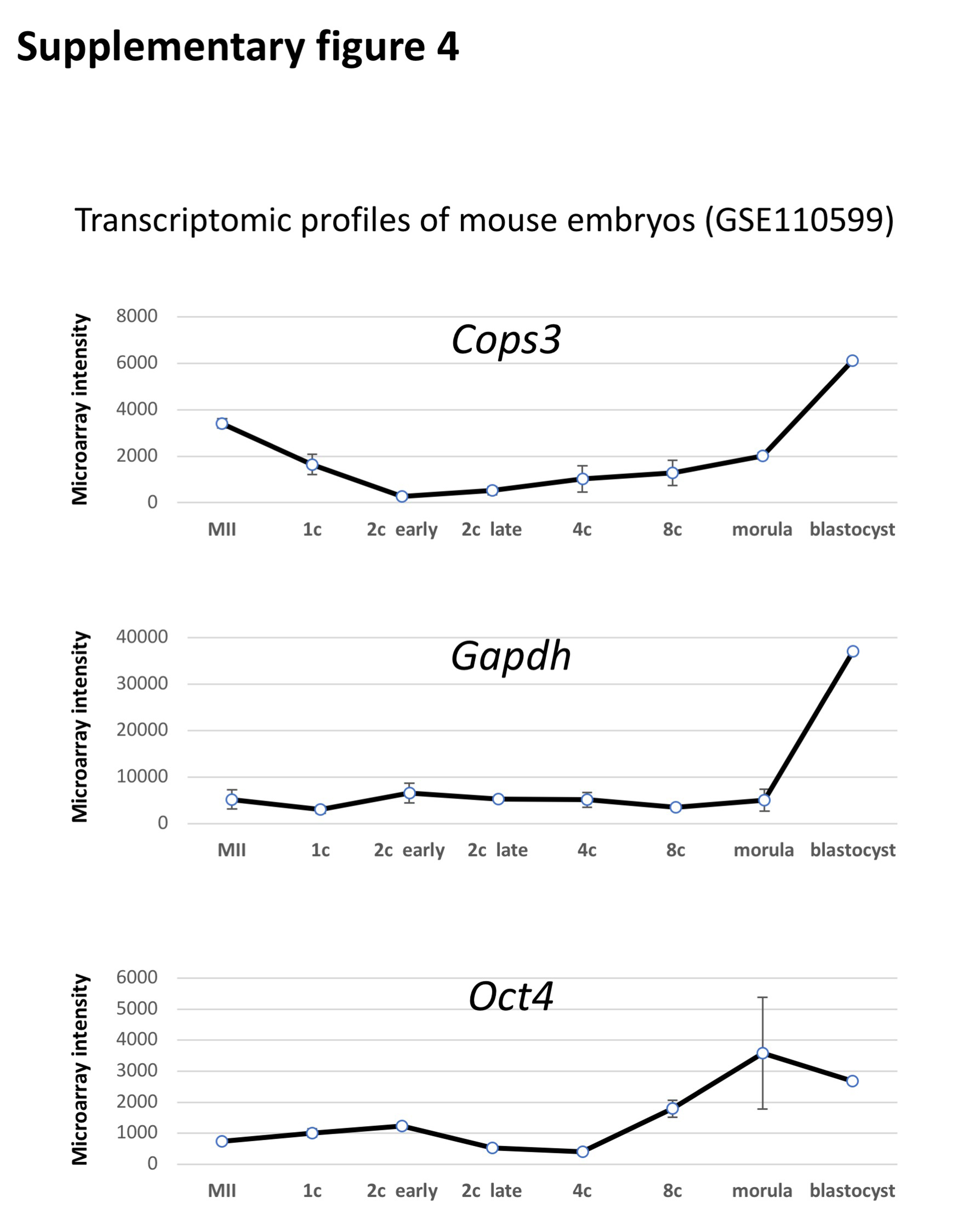

Supplement: Casser_et_al_Supplementary_figure_4_Aug12_2019_gaz051 [file casser_et_al_supplementary_figure_4_aug12_2019_gaz051.jpeg]
